# Predictive model for laser-induced tissue necrosis with immunohistochemistry validation

**DOI:** 10.1117/1.BIOS.1.2.025003

**Published:** 2024-08-05

**Authors:** J. Junior Arroyo, Arunima Sharma, Jiaxin Zhang, Muyinatu A. Lediju Bell

**Affiliations:** a Johns Hopkins University, Department of Biomedical Engineering, Baltimore, Maryland, United States; b Johns Hopkins University, Department of Electrical and Computer Engineering, Baltimore, Maryland, United States; c Johns Hopkins University, Department of Computer Science, Baltimore, Maryland, United States

**Keywords:** photoacoustic imaging, laser safety, numerical simulation, microscopy, single-cell segmentation, necrosis

## Abstract

**Significance:**

Photoacoustic imaging holds promise to provide critical guidance in surgical interventions, but its widespread use is challenged by the absence of applicable safety guidelines across diverse target tissues. The biosafety of this technology is primarily associated with the risk of necrosis generation, which is an irreversible thermal effect that can result from prolonged, high-energy laser applications.

**Aim:**

We introduce the first known numerical simulation approaches to assess laser-induced necrosis in liver tissue and present a novel microscopy analysis framework to validate performance.

**Approach:**

Our simulation methods integrate Monte Carlo simulations of laser-tissue interaction with the COMSOL interface, model local tissue heating, and predict associated tissue damage to quantify the percentage of tissue necrosis resulting from laser application. Our initial predictions are based on 30 and 73 mJ mean laser energies, laser irradiation times of 1, 10, and 20 min, and a 750 nm laser wavelength. Empirical validations with *in vivo* porcine liver exposed to a mean laser energy of 73 mJ and 750 nm laser wavelength were performed based on H&E and cleaved Caspase-3 immunohistochemistry (IHC) results. Simulation results from the lower 30 mJ laser energy were additionally cross-referenced with previous qualitative H&E-based reports.

**Results:**

Negligible tissue damage was observed with necrosis predictions ≤15.05%, damage thresholds were determined to be within the 15.05% to 66.23% necrosis prediction range, and necrosis predictions deviated from quantitative IHC results by 0.01% to 8.1%.

**Conclusions:**

We successfully demonstrated an *in silico* alternative to the otherwise time-consuming and expensive empirical assessments that would be required to create tissue-specific laser safety guidelines. The presented methods have the potential to be translated to multiple tissues and additional laser properties.

Statement of Discovery We introduce the first-known study demonstrating a unified theoretical, computational, and experimental approach to determine laser safety for biological tissues other than skin or eyes.

## 1 Introduction

Photoacoustic imaging is a beneficial technology that integrates the advantages of ultrasound and optical imaging. Structures with high optical absorption coefficients absorb light, leading to a localized temperature rise that generates thermal expansion. This expansion results in tissue relaxation and the subsequent isotropic release of acoustic waves, which are ultimately detected by ultrasound sensors. The broad range of targets that can be effectively imaged using photoacoustic imaging has been highlighted in many reports, positioning it as a valuable tool for surgical guidance.^1^^–^^4^

Laser safety is an important biohazard consideration to ensure patient safety during photoacoustic interventional guidance. The maximum permissible exposure (MPE) is defined by the American National Standards Institute, which provides guidelines to determine the maximum allowable pulse energy per unit area to prevent any adverse biological effects. However, these guidelines currently focus solely on eyes and skin. The MPE for skin is commonly used as a reference for internal tissues during surgical and interventional guidance testing,^1^ but this assumption can unnecessarily limit the MPE.^1^^,^^5^

Experiments were previously conducted to determine appropriate parameters to minimize and control the risk of tissue damage in specific internal tissues, such as the liver^5^^,^^6^ and heart.^7^ Kempski et al.^6^ investigated suitable laser energies for *in vivo* photoacoustic imaging of porcine hepatic blood vessels, demonstrating that a minimum energy of 30 mJ ($153\;\mathrm{mJ}/{\mathrm{cm}}^{2}$ fluence), emitted at a wavelength of 750 nm, was necessary for optimal visualization, even though this energy exceeded the MPE limit for skin and revealed necrosis, hemorrhage, and inflammation with the associated exposure time of 80 min. Building on these findings, Huang et al.^5^ investigated the impact of ∼30  mJ of laser energy on 35 swine liver specimens with exposure times of 1, 10, and 20 min and demonstrated the absence of necrosis under these shorter time durations. Similarly, Graham et al.^7^ delivered $379.2\;\mathrm{mJ}/{\mathrm{cm}}^{2}$ fluence to cardiac tissue using a wavelength of 750 nm for a time duration of 23 min, without any observed pathological tissue changes due to irradiation, despite exceeding the $25.6\;\mathrm{mJ}/{\mathrm{cm}}^{2}$ MPE for skin. In each case, the pulse duration was 5 ns and the pulse repetition rate was 10 Hz.^5^^–^^7^

While these previous studies support the conclusion that safety limits for skin are not suitable when applied to internal tissue, the qualitative nature of the histological assessment categorizes tissue conditions into five distinct levels: not present, minimal, mild, moderate, and severe. Retrospective studies have demonstrated that assigning categorical grades to tissue conditions can lead to substantial inter- and intra-observer variability, making the process highly subjective.^8^^–^^11^ In addition, the combined processes of tissue processing, digitization, and pathologist reading can collectively be time consuming and resource-intensive, considering the requirements for stains, fixatives, slides, histology equipment, and knowledgeable personnel who can manage and allocate these resources. We hypothesize that theoretical modeling and *in silico* validations have the potential to provide a more reliable, repeatable, less time-consuming, and less resource-intensive approach to determine tissue-specific laser safety guidelines.

This paper presents a comprehensive theoretical, *in silico*, and experimental assessment of laser-induced tissue necrosis on swine liver samples, with three primary contributions. First, we rely on theoretical equations to present a novel simulation framework that predicts the percentage of tissue necrosis based on laser energy, beam diameter, wavelength, and exposure time. Second, we validate simulation predictions by focusing on the impact of delivering a mean laser energy of ∼73  mJ ($372\;\mathrm{mJ}/{\mathrm{cm}}^{2}$ fluence) to *in vivo* liver tissue, with irradiation durations of 1, 10, and 20 min, a wavelength of 750 nm, 5 ns pulse duration, and 10 Hz pulse repetition frequency. These choices are based on prior observations of necrosis occurring at the same laser parameters (i.e., wavelength, pulse duration, and pulse repetition frequency) with 20 to 40 mJ laser energy (102 to $204\;\mathrm{mJ}/{\mathrm{cm}}^{2}$ fluence) and 80 min laser duration.^6^ In addition, damage was absent for the same laser wavelength with ∼30  mJ energy ($153\;\mathrm{mJ}/{\mathrm{cm}}^{2}$ fluence) with a laser irradiation duration of 20 min.^5^ These prior results indicate that we can expect a spectrum of tissue necrosis outcomes for the same wavelength with a factor of ∼1.8 to 3.7 greater energy than 20 to 40 mJ (i.e., 73 mJ) and 1 to 20 min irradiation times. Third, we introduce a method to quantitatively assess tissue necrosis percentage from digitized immunohistochemistry sections, which is a significant departure from previous qualitative approaches. This transition provides an unambiguous interpretation of tissue conditions, eliminates the reliance on grading scales, and facilitates an alternative predictive modeling approach.

## 2 Methods and Materials

### 2.1 Simulation Framework for Thermal Damage Estimation

To determine damage, tissue-photon interactions were first modeled during laser energy delivery using three-dimensional (3D) Monte Carlo simulations.^12^ These simulations were performed with the optical properties summarized in Table 1, which are specific to swine liver tissue characteristics at a wavelength of 750 nm, for later comparisons with experimental results. The optical absorption coefficient dictates the depth of photons penetrating tissue prior to being absorbed. The scattering coefficient dictates how the tissue scatters photons outside of the laser beam, and the anisotropic factor determines the amount of forward direction propagation retained after scattering. These parameters were modeled within a $20\times 20\times 20\;{\mathrm{mm}}^{3}$ homogeneous porcine liver block. A 5 mm-diameter laser source touching one surface of this cubic volume irradiated energy for 1, 10, and 20 min with an optical wavelength of 750 nm. The output of the 3D Monte Carlo simulations is a spatial distribution of normalized energy density, which can be used to assist with defining the optical laser delivery as a heat source.

**Table 1:** Optical and thermodynamic parameters utilized in simulations.

	Parameter	Value	Units	Ref.
Optical	Absorption coefficient	0.1	1/mm	13
	Scattering coefficient	6.14	1/mm	13
	Anisotropic factor	0.9	—	13
Thermodynamic	Blood perfusion rate ($\omega_{b}$)	0.0175	1/s	14
	Blood specific heat ($c_{b}$)	3617	J/(kg·K)	15
	Blood density ($\rho_{b}$)	1050	${\mathrm{kg}/m}^{3}$	15
	Arterial blood temperature ($T_{b}$)	310.15	K	16
	Frequency factor (A)	$5.51\times 10^{41}$	1/s	17
	Activation energy (ΔE)	$2.77\times 10^{5}$	J/mole	17

To define a heat source, heat conduction was modeled using COMSOL Multiphysics 6.1.^18^ A homogeneous cubic tissue with the same size as the porcine liver block described above (i.e., $20\times 20\times 20\;{\mathrm{mm}}^{3}$) was also modeled considering three domains. In the first domain, which constitutes the surface directly exposed to the 5 mm-diameter laser beam, triangular elements with a maximum size of 2 mm were used. In the second domain, which covers the surface not exposed to the laser beam, triangular elements with a maximum size of 3 mm were used. In the third domain, which is the remainder of the modeled volume, quadrilateral elements with a maximum size of 3 mm were used. All boundaries were kept at constant temperature except for the liver surface. Heat exchange in the liver surface was based on free convection in air, resulting from the liver exposure to the environment. This convective effect influences the local temperature dynamics induced by the laser.

To monitor the tissue temperature over time and ultimately predict local tissue damage, the Time-Dependent Bioheat Transfer interface within COMSOL Multiphysics 6.1 was used to solve the Pennes Bioheat Transfer equation. This equation describes the time-dependent biological heat transfer to model hyperthermia processes in perfused tissue, as follows:

$$\rho c_{p}\frac{\partial T}{\partial t}+\rho c_{p}\cdot \nabla T+\nabla \cdot q=Q+\rho_{b}c_{b}\omega_{b}(T_{b}-T)+Q_{\text{met}},$$ (1)

where q is the heat flux density, defined as

q=−k∇T. (2)

The remaining variables are based on properties of the liver (i.e., ρ, T, $c_{p}$, k), associated blood (i.e., $\rho_{b}$, $T_{b}$, $c_{b}$, $\omega_{b}$), and heat sources (i.e., Q, $Q_{\mathrm{met}}$). In particular, ρ and $\rho_{b}$ are the liver and blood density, respectively; T and $T_{b}$ are the temperatures of the liver and blood, respectively; $c_{p}$ and $c_{b}$ are the specific heat capacities of the liver and blood, respectively (representing the energies required to raise T and $T_{b}$, respectively, by one unit of temperature per unit mass); k is the thermal conductivity (which quantifies the ability of the liver to conduct and retain heat); $\omega_{b}$ is the blood perfusion rate (which accounts for the circulation of blood through an *in vivo* vascularized liver); Q and $Q_{\mathrm{met}}$ are the laser and metabolic heat sources, respectively; and t is the time. The values of $\omega_{b}$, $c_{b}$, $\rho_{b}$, $T_{b}$ employed in our study are reported in Table 1 for the porcine liver, whereas ρ, $c_{p}$, and k were considered to be temperature-dependent, based on the details reported by Rossmann and Haemmerich.^19^ The impact of body heat production on temperature was not considered due to the lack of sufficient literature values for the porcine liver (i.e., $Q_{\mathrm{met}}=0$), and Q in Eq. (1) was obtained as follows:

Q=P·Energy Density·ONOFF, (3)

where P is the laser power (converted from the associated laser energy per pulse duration), Energy Density is the normalized energy density distribution from the Monte Carlo simulations described above, and ONOFF is a trigger that periodically turns the laser on and off to replicate a pulsed laser source. Energy Density was exported from MATLAB as a four-column csv file depicting 3D coordinates and the density value, then imported into COMSOL. The ONOFF function forms a square wave when plotted over time. In our implementation, a pulse energy of 73 mJ ($371.79\;\mathrm{mJ}/{\mathrm{cm}}^{2}$ fluence) was delivered using a 10 Hz pulse repetition frequency with a 5 ns pulse duration. Consequently, the laser power was $1.46\times 10^{7}\;W$. With one pulse emitted every 0.1 seconds (i.e., 10 Hz pulse repetition frequency), the ONOFF function takes a value of 1 for the first 5 ns and then reverts to 0 during each interval. As a result, Energy Density from Monte Carlo simulations was modeled to be delivered for 5 ns every 0.1 s. For comparison with our previous report,^5^ this process was repeated after decreasing the energy to 30 mJ (i.e., laser power $6\times 10^{6}\;W$) while preserving pulse duration and pulse repetition frequency.

A domain point probe was placed at the irradiated surface in the COMSOL simulations to monitor T(t) in Eq. (1), and the associated degree of tissue injury, α(t), was obtained by solving the following differential equation of the Arrhenius Kinetics model:

$$\frac{\partial \alpha }{\partial t}=(1-\alpha {)}^{n}{\mathrm{Ae}}^{\left(\Delta E/RT\right)},$$ (4)

where n is the polynomial order of the Arrhenius equation (specifically, n=3 was empirically determined to best match simulation outcomes with experimental results), R is the universal gas constant, A is the frequency factor that refers to the likelihood of damage-inducing events occurring within a time frame, and ΔE is the activation energy that represents the energy threshold that must be exceeded to induce tissue damage. The values of A and ΔE employed in our study are associated with the onset of irreversible thermal damage in swine liver and are reported in Table 1. The fraction of necrotic tissue, $\theta_{d}$, was then computed from Eq. (4) as follows:

$$\theta_{d}=\min(\max(\alpha ,0),1).$$ (5)

Finally, Eq. (5) was multiplied by 100% to report the percentage of necrotic tissue predicted by simulations.

### 2.2 Experimental Procedures to Assess Necrosis

**Fig. 1:**
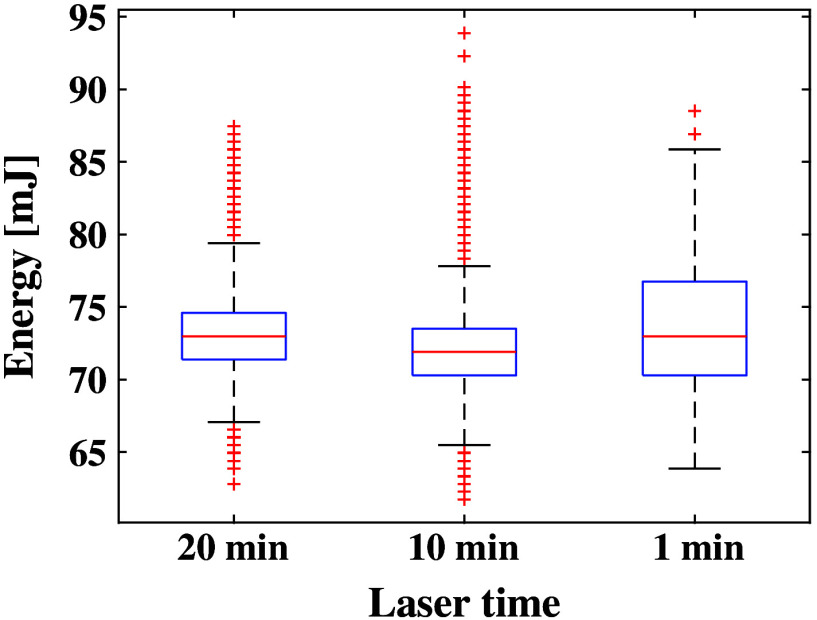
Distribution of laser energy delivery per laser application duration, shown as box-and-whisker plots. The median energy is indicated by the red horizontal line, the interquartile range is indicated by the top and bottom bounds of each box, and the maximum and minimum values (excluding outliers, appearing as red datapoints and defined as values >1.5 times the interquartile range) are indicated by the lines extending from each box.

A 5 mm-diameter fiber bundle was connected to a Phocus Mobile laser containing an internal power meter (Opotek, Carlsbad, California) to deliver output desired energies to the surface of exposed *in vivo* liver tissue. The laser delivered 5 ns pulse widths at a pulse repetition frequency of 10 Hz. To monitor and compensate for known fluctuations in energy and maintain a desired sliding-average pulse-to-pulse energy range (with a 50-pulse window) of 71 to 75 mJ, the internal power meter of the laser and a custom command-line interface were employed.^5^ In particular, a calibration between the internal power meter and an external power meter was performed prior to the start of the procedure, and the internal power meter was used to record and adjust real-time laser energies throughout the duration of the laser application. Figure 1 shows the pulse-to-pulse energy measurements during different laser application times. The interquartile ranges of delivered energy was 70.29 to 76.75 mJ, 70.28 to 73.50 mJ, and 71.37 to 74.59 mJ for the 1, 10, and 20 min time durations, respectively. The mean energy is reported in Table 2.

**Table 2:** Laser energy delivery positions and duration.

	Lobe region	Mean energy (mJ)	Duration (min)
Sample 1	Left lateral lobe (cranial)	73.0	20
Sample 2	Left lateral lobe (center)	72.5	10
Sample 3	Left lateral lobe (caudal)	73.6	1

**Fig. 2:**
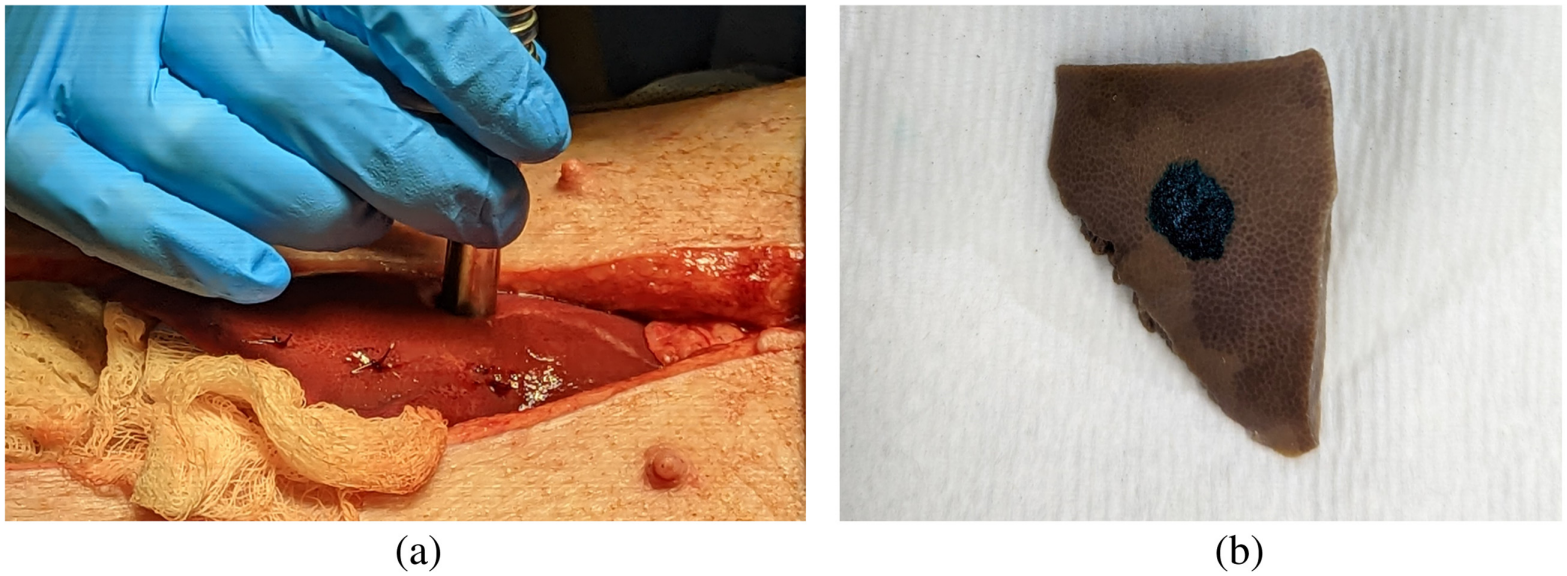
The marking strategies to identify irradiated areas on the swine liver samples included (a) sutures before laser application and (b) tissue dye after liver lobe resection (and prior to sample fixation).

A laparotomy was performed on the abdomen of a female Yorkshire swine (36 to 40 kg) to access and expose the left lateral liver lobe. Laser energy was applied to three positions on the surface of the left lateral lobe for 1, 10, and 20 min, creating three samples for analysis, as detailed in Table 2. The three differences in irradiation time are expected to induce minimal, moderate, and severe necrosis, based on our previous results.^5^ The irradiated positions were marked by first placing a line of suture perpendicular to and 3 cm away from each intended laser application site. The three suture lines are observable in Fig. 2(a). After laser application, the irradiated regions were directly inked using a tissue marking dye. After euthanasia, the entire left lateral liver lobe was removed from the abdomen, irradiated regions were excised [Fig. 2(b)], and immediately fixed in a 10% formalin solution. This study was approved by the Johns Hopkins University Institutional Animal Care and Use Committee.

### 2.3 Qualitative Histopathology Assessment

To provide baseline comparisons with previous qualitative reports, the excised samples described in Sec. 2.2 were embedded in paraffin, then placed in a microtome with the irradiated surface oriented approximately parallel to the microtome blade. Each sample was sectioned into 250 slides with a section thickness of 4  μm. Out of the collection of 250 sections, one section was selected for staining with Hematoxylin and Eosin (H&E), which was extracted from a depth of 928  μm, to guarantee the representation of the entire cross-section of the tissue sample. As H&E was previously used to qualitatively identify the presence of necrosis, hemorrhage, and inflammation,^5^ the samples described above were similarly assessed for these three pathological features (i.e., absent, minimal, mild, moderate, or severe).

### 2.4 Qualitative Immunohistochemistry Assessment

Out of the collection of 250 sections per liver sample described in Sec. 2.3, a representative subset consisting of one per every 10 sections (i.e., 40  μm spacing) was selected for immunohistochemistry (IHC) staining with an antibody specific to cleaved Caspase-3, diluted at a ratio of 1:1000. In addition, three additional sections near the surface of each sample, separated by 8  μm per section, were stained with IHC to provide mean and standard measurements for comparison to surface simulation results. Cleaved Caspase-3 was the antibody chosen to identify necrosis biomarkers because of its well-established role in apoptosis, a programmed cell death mechanism. This choice was additionally motivated by our preliminary qualitative observations, which revealed a positive correlation between cleaved Caspase-3 and the identification of necrotic areas resulting from prolonged laser irradiation.

**Fig. 3:**
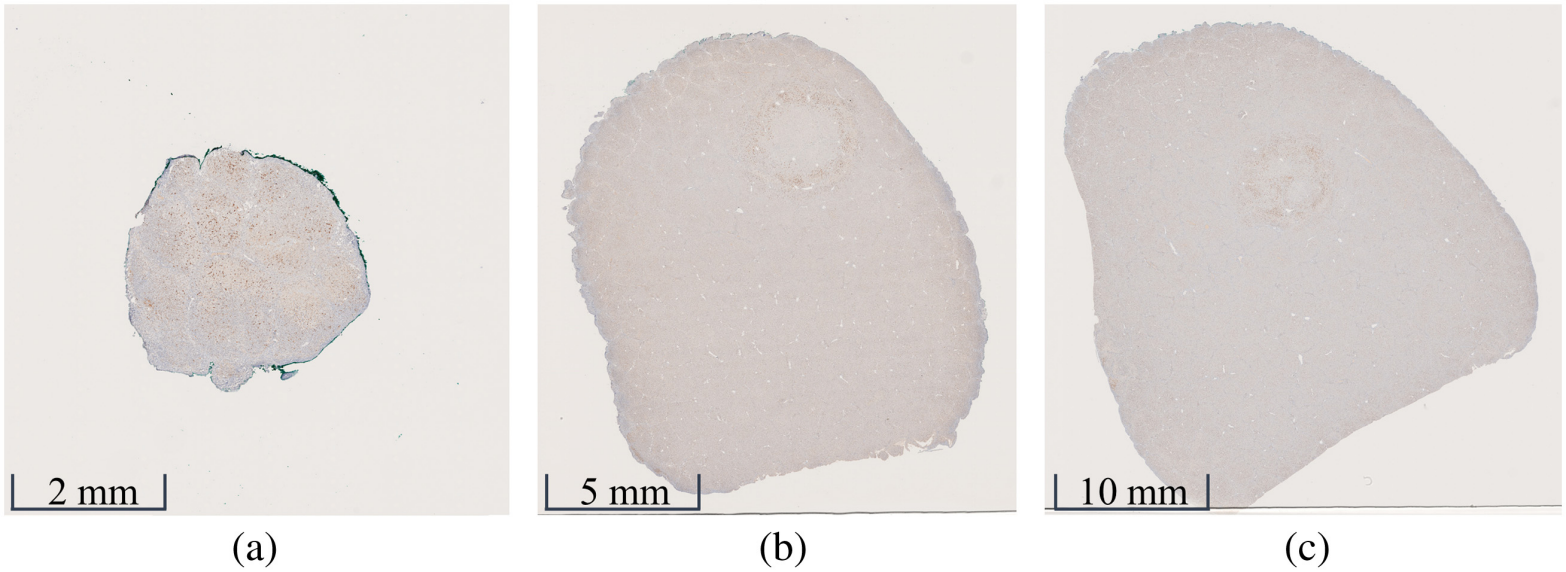
Digitized immunohistochemistry sections extracted at depths of (a) 0  μm, (b) 480  μm, and (c) 960  μm from a porcine liver sample irradiated for 10 min. The tissue section size increases with depth due to the tissue curvature.

To systematically characterize our qualitative observations, individual IHC sections were digitized at 40× magnification using a Hamamatsu NanoZoomer S210 to generate NDPI files. The NDPITools Plugin Bundle of ImageJ^20^ was used to extract the content of the NDPI file by dividing each digitized IHC section into a mosaic of adjacent JPEG images, encoding color information using Red-Blue-Green (RGB) channels. NDPITools automatically selected mosaic dimensions that were powers of two in each image dimension, resulting in individual JPEG image sizes ranging 700 to 1300×700 to 1300 pixels, based on a 4MB storage restriction. When these multiple JPEG images were spatially arranged, aligned, and stitched together, the IHC section was accurately reconstructed. As the area of the digitized tissue sections generally increased with depth (see Fig. 3), due to the curved surface of the samples, the number of JPEG images that composed each mosaic varied. For example, the sizes of the IHC sections in Figs. 3(a)–3(c) are 30,720×30,976, 76,800×81,664, and 103,680×101,376 pixels, respectively, which were decomposed into 32×32, 128×64, and 128×128 mosaics, respectively, each comprising individual JPEG images sized 960×968, 1200×638, and 810×792  pixels, respectively (i.e., 1024, 8192, and 16,384 individual JPEG images, respectively). Overall, the number of individual JPEG images from the IHC sections associated with the 1, 10, and 20-min laser irradiation samples was 16,384 (constant for each section), 1024 to 16,384, and 2048 to 16,384, respectively.

**Fig. 4:**
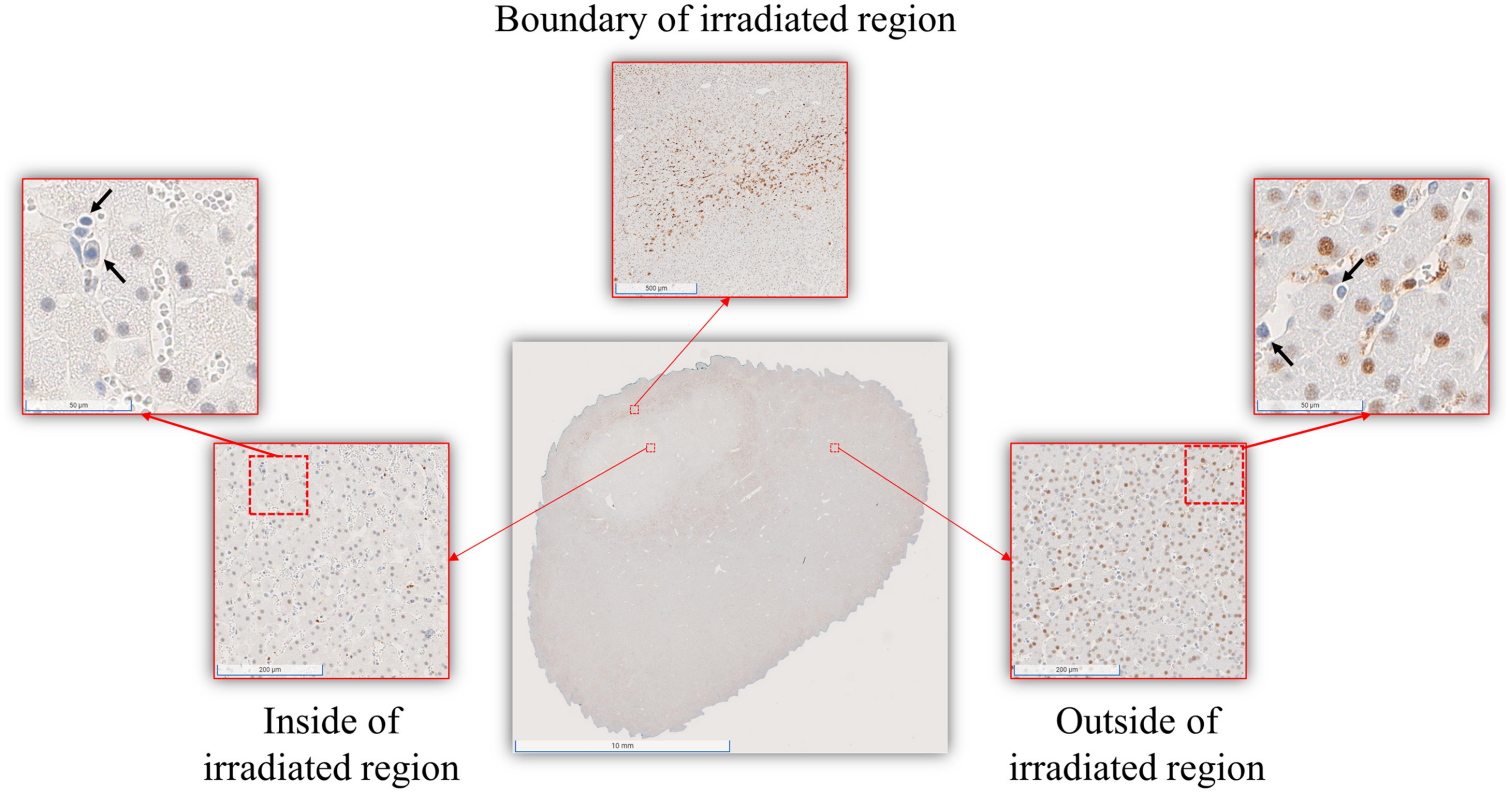
Visual inspection of a representative digitized IHC section. Blue cells are pointed out by arrows. The overall cell density was higher outside than inside the irradiated area. The boundary of the irradiated area presented a high concentration of brown cells.

To qualitatively identify the presence of biomarkers at the cellular level, we conducted a visual inspection of the digitized IHC sections, revealing two distinct types of cells: (1) cleaved Caspase-3-positive cells stained in intense brown and (2) cleaved Caspase-3-negative cells stained in intense blue, which will be referred to as brown and blue cells, respectively. The distribution of these cells presented four notable characteristics, which are shown in Fig. 4. First, a decrease in cell density was observed inside the irradiated region compared to non-irradiated areas. Second, blue cells (examples denoted with arrows in Fig. 4) were present both inside and outside visibly irradiated areas. Third, brown cells were predominantly observed surrounding the irradiated region. Fourth, the number of brown cells detected within the irradiated area was inversely proportional to the irradiation time.

To segment blue and brown cells from the digitized IHC sections, an Attention U-Net^21^ was employed. The architecture of the Attention U-Net consisted of four encoder layers, four decoder layers, and four attention gates. This network was trained using the Adam optimizer, a batch size of four samples, and standard data augmentation techniques (i.e., flip, elastic transformation, grid distortion, and optical distortion). Early stopping with a patience of 10 was employed to avoid overfitting. The Attention U-Net was trained with images from the current dataset. The network dataset contained 50 images along with their manual segmentations, with an 80%–20% training–testing set split, and 20% of the training set forming the validation set. Performance was quantified using the Dice similarity coefficient (DSC) as the main metric, following the guidelines of evaluation metrics for medical image segmentation.^22^ In addition, the associated intersection over union (IoU), recall, and precision were calculated. The weights obtained from this training process were stored and utilized for cell segmentation in each JPEG image. This segmentation was performed using Python 3.9.15 in a Jupyter Notebook.

Following cell segmentation, individual cells were analyzed for classification into blue or brown. A cell was categorized as brown if its RGB components satisfied the condition 60≤R≤210, G≤151, and B≤130. A cell was categorized as blue if its RGB components satisfied the condition 150≤R≤186, G≥155, and B≥160. These specific thresholds were empirically chosen based on the analysis of multiple images from our dataset. To reconstruct a section segmentation of blue and brown cells, the segmentation masks obtained from individual JPEG images were spatially arranged, aligned, and stitched together to achieve the same size and relative orientations that existed prior to creating the mosaic. This image processing was performed using MATLAB R2023a (Mathworks, Natick, Massachusetts) software.

### 2.5 Quantitative Necrosis Mapping

To quantify necrosis as a percentage based on the digitized IHC sections, the observed spatial distributions and qualitative characteristics of blue and brown cells noted in Sec. 2.4 were modeled as an exponential decay, as follows:

$$\text{Necrosis}=\Gamma e^{-k\rho }\times 100\%,$$ (6)

where Γ and ρ represent the fractions of the areas of blue or brown cells, and k=100 enables us to define ρ as a fraction rather than the percentage that kρ represents. The fractions were defined as

$$\Gamma =\frac{\text{blue cell area}}{\text{blue cell area}+\text{brown cell area}},$$ (7)

and

$$\rho =\frac{\text{brown cell area}}{\text{total patch area}},$$ (8)

where the total patch area is 200  μm×200  μm, which was selected to display ∼20 to 200 cells per patch (i.e., the cells were ∼5 to 10  μm in diameter). Smaller-sized patch areas captured regions devoid of blue cells or completely covered by clusters of brown cells, while larger sizes compromised the intent of this local calculation. When there are no brown cells within the irradiated region, the model presented in Eqs. (6)–(8) successfully achieved Γ=1, ρ=0, and 100% necrosis, with a lower necrosis percentage achievable when brown cells are present. The multiplicative exponential term, $e^{-k\rho }$, was introduced to proportionally scale the fraction of blue cells (i.e., Γ) present by the local density of brown cells (i.e., ρ). This exponential term produced a steep change in the % necrosis near the boundary of the irradiated region.

### 2.6 Registration of Digitized IHC Sections and Necrosis Maps

To place the independent two-dimensional (2D) serial digitized IHC sections and necrosis maps in a common coordinate frame for volumetric reconstruction, a rigid registration was first performed between digitized IHC sections. This registration enabled the computation of deformation maps for each section. The deformation maps were then used to warp the corresponding necrosis maps.

To perform digitized IHC sections registration, a section in the middle of the stack was selected as the reference section based on overall appearance, optimal contrast, and depiction of the entire irradiated area. This reference section selection is critical to avoid error propagation and ensure accurate reconstruction results. We purposely avoided selecting the first section of the stack as the reference, which can introduce reconstruction artifacts such as skewed or helical volumes.^23^ The carefully selected reference section was then registered with its two direct neighboring sections, and the registration process was performed in both forward and backward directions (i.e., pairwise registration until the last and first sections, respectively). During this automatic registration process, geometric features were extracted from the IHC sections with the salient feature being the brown cell region surrounding the irradiated area. After registration, the IHC sections were transformed from the RGB to the $L^{*}a^{*}b$ color scale to highlight the irradiated area and assist with automatic feature extraction. Each pair of adjacent IHC sections evaluated produced a deformation map, which was then used to warp the corresponding necrosis map.

**Fig. 5:**
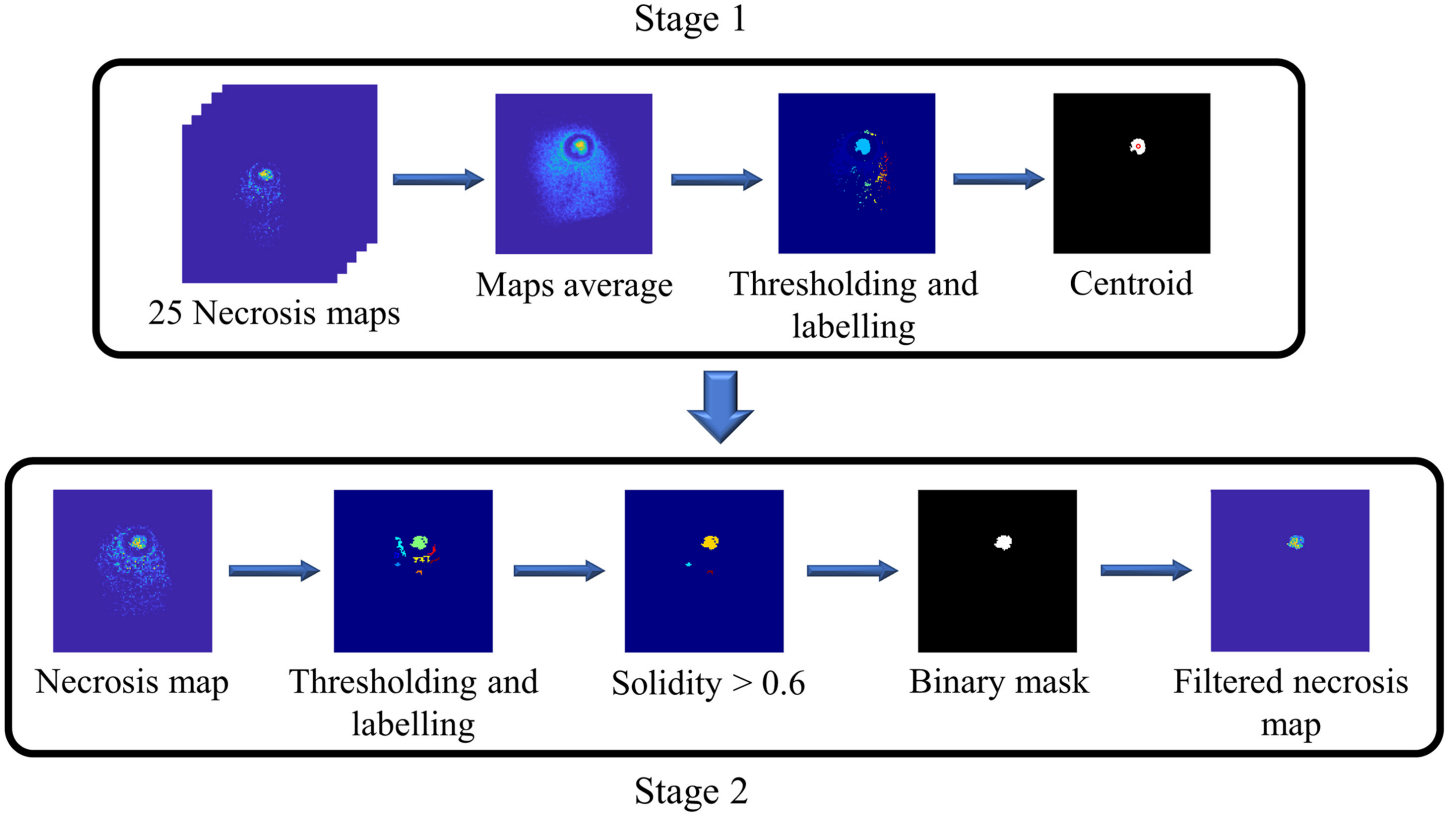
Examples of intermediate and final outcomes of the two-stage artifact filtering process.

To remove artifacts from the registered necrosis maps [i.e., necrotic areas appearing in addition to the irradiated region, which are artifacts introduced by Eq. (6)], we implemented a two-stage filtering process consisting of identifying the centroid of necrosis from all maps in a volume, followed by filtering individual maps in the volume, as illustrated in Fig. 5. In Stage 1, the 25 registered necrosis maps were first averaged to yield an average necrosis map, with the maximum value consistently located within the irradiated area. The histogram of the average necrosis map exhibited a quadrimodal distribution, where four peaks represented the background, low, intermediate, and high necrosis percentage values in increasing order of pixel intensity. Using Otsu’s method,^24^ three threshold values based on pixel intensities of the average necrosis map were computed. As the irradiated region consistently presented regions of intermediate and high necrosis values, the second threshold value was used, converting the average necrosis map into a binary image. Each binary object within the binary image was assigned a non-negative integer as a label. The binary object encompassing the maximum value of the average necrosis map was preserved. The centroid of the selected binary object was computed and utilized to filter individual necrosis maps in Stage 2, which was initiated by converting each individual map to binary objects using the Otsu’s method described in Stage 1. Non-integer numbers were assigned as labels to individual binary objects for easy reference during image processing. Next, the solidity (i.e., a measure of compactness and convexity) of each individual object was computed. Objects with a solidity lower than 0.6 were discarded, as they were unlikely to represent the circular necrosis area. The distances between the centroid of each remaining binary object and the centroid computed in Stage 1 were calculated, and the lowest distance was identified as corresponding to the necrotic area, resulting in a filtered binary mask. The initial individual necrosis map was multiplied by the filtered binary mask to remove artifacts. The 25 filtered binary masks were used to generate a necrosis volume. These masks have a spatial separation of 40 um, resulting from staining one out of every 10 sections using immunohistochemistry (as described in Sec. 2.4). Therefore, linear interpolation was performed to recover the masks from the missing sections and complete the volume. This image processing was performed using MATLAB R2023a (Mathworks, Natick, Massachusetts) software.

### 2.7 Comparisons between Simulated and Experimental Results

To compare simulation predictions and experimental results, we first consider that a direct comparison is challenging due to the liver samples exhibiting a curved surface, whereas the simulated tissue model was designed with a flat surface. To address this geometric disparity, we selected the most superficial IHC section that fully displayed the irradiated region and its adjacent non-irradiated area. Following this protocol, the sections selected for comparison were extracted at depths of 0, 280, and 160  μm for the 1, 10, and 20-min irradiation times, respectively. We then calculated the absolute error in percent necrotic tissue between simulated predictions (Sec. 2.1) and experimental results for the selected sections (Sec. 2.5), as a function of irradiation time. To strengthen the validity of our findings, five total sections at depths 280 to 320  μm and 160 to 200  μm from the 10- and 20-min samples, respectively, were each combined to report a mean and standard deviation per sample for additional comparison to the corresponding simulation results.

## 3 Results

### 3.1 Simulated Necrosis Predictions

**Fig. 6:**
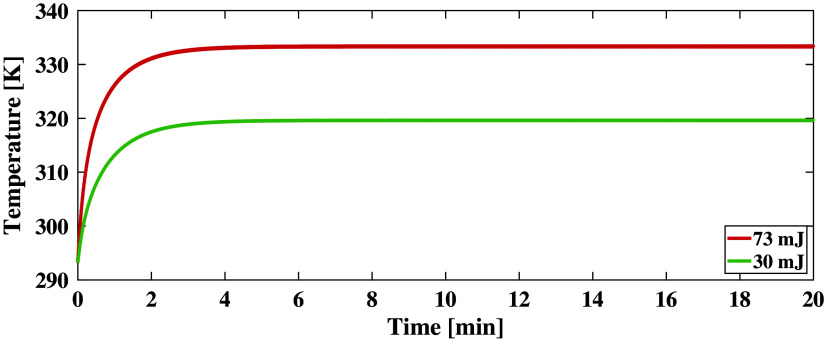
Time-resolved temperature prediction at the tissue-laser interface for a simulated liver exposed to 73 and 30 mJ laser energy.

Figure 6 shows simulated temperature as a function of time for the delivery of 30 and 73 mJ laser energies. The initial temperature of each sample was 293.15 K (because laparotomy typically exposes the liver to the environment). The temperature profile for the 73 mJ laser energy exhibited three phases. During the first phase (0 to 0.34 min), there was a rapid linear temperature increase in the liver, up to 315.15 K where the inactivation of vital enzymes occurs.^25^ In the second phase (0.34 to 3.88 min), the temperature continued escalating at a lower rate, eventually reaching a maximum temperature of 333.15 K, which is the threshold temperature for protein denaturation.^25^ In the third phase, the temperature stabilized and was maintained at 333.44 K. In contrast, the temperature profile associated with the 30 mJ laser energy has two phases, with the first stage (0 to 4.94 min) characterized by a gradual temperature increase (up to 319.56 K, which surpasses the temperature known to inactivate vital enzymes^25^) and the second phase characterized by temperature stabilizing at 319.66 K.

**Fig. 7:**
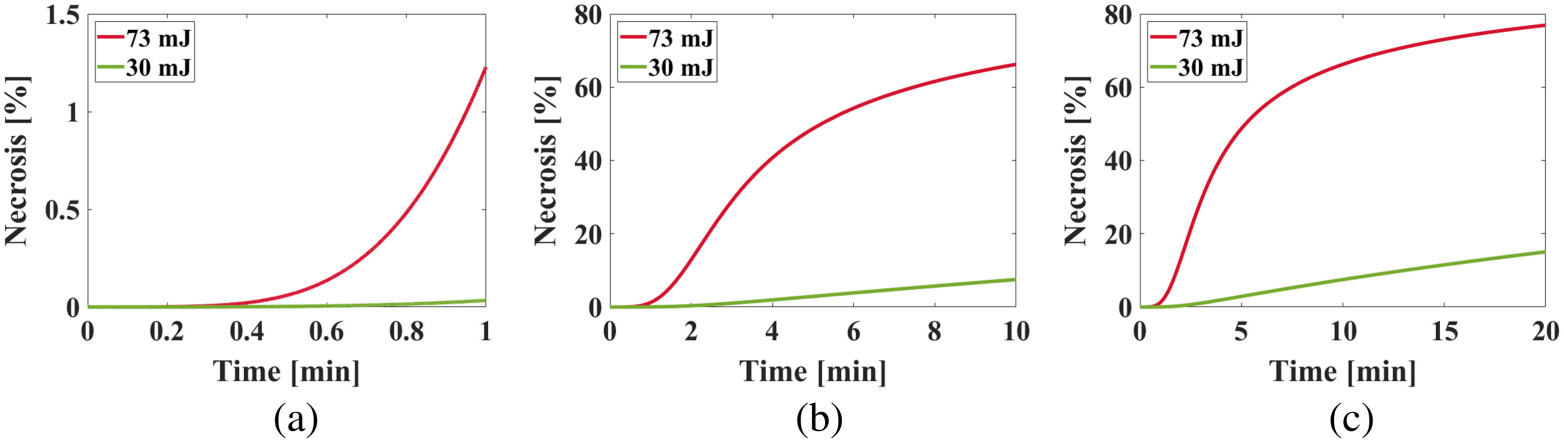
Time-resolved necrosis percentage prediction at the tissue-laser interface for simulated liver samples irradiated for (a) 1 min, (b) 10 min, and (c) 20 min.

Figure 7 shows the time-resolved predictions for necrosis percentage at the tissue-laser interface for different exposure times and laser energies. The induced damage slowly progressed during the first minute of irradiation, resulting in necrotic tissue percentages of 0.03% and 1.23% for 30 and 73 mJ, respectively. Extending the laser exposure to 10 min led to necrotic tissue percentages of 7.49% and 66.23% for 30 and 73 mJ, respectively. Following 20 min of laser irradiation, the necrotic tissue percentage escalated to 15.05% and 76.84% for 30 and 73 mJ, respectively. Table 3 summarizes these simulation results.

**Table 3:** Comparison of simulated predictions and qualitative histopathological grading of necrosis in swine liver samples irradiated with ∼30 and 73 mJ laser energy for 1, 10, and 20 min.

Energy	Laser time (min)	Necrosis prediction (%)	Necrosis grading
30 mJ	1	0.03	− −
	10	7.49	− −
	20	15.05	− −
73 mJ	1	1.23	− −
	10	66.23	+
	20	76.84	+++

Necrosis is graded as absent (− −), minimal (−), mild (+), moderate (++), and severe (+++) based on H&E stains. Experimental necrosis gradings for ∼30  mJ energy (obtained with a laser pulse duration of 5 ns and 10 Hz pulse repetition frequency) were derived from previously reported results from 35 liver samples.^5^

### 3.2 Empirical Necrosis Assessment

**Fig. 8:**
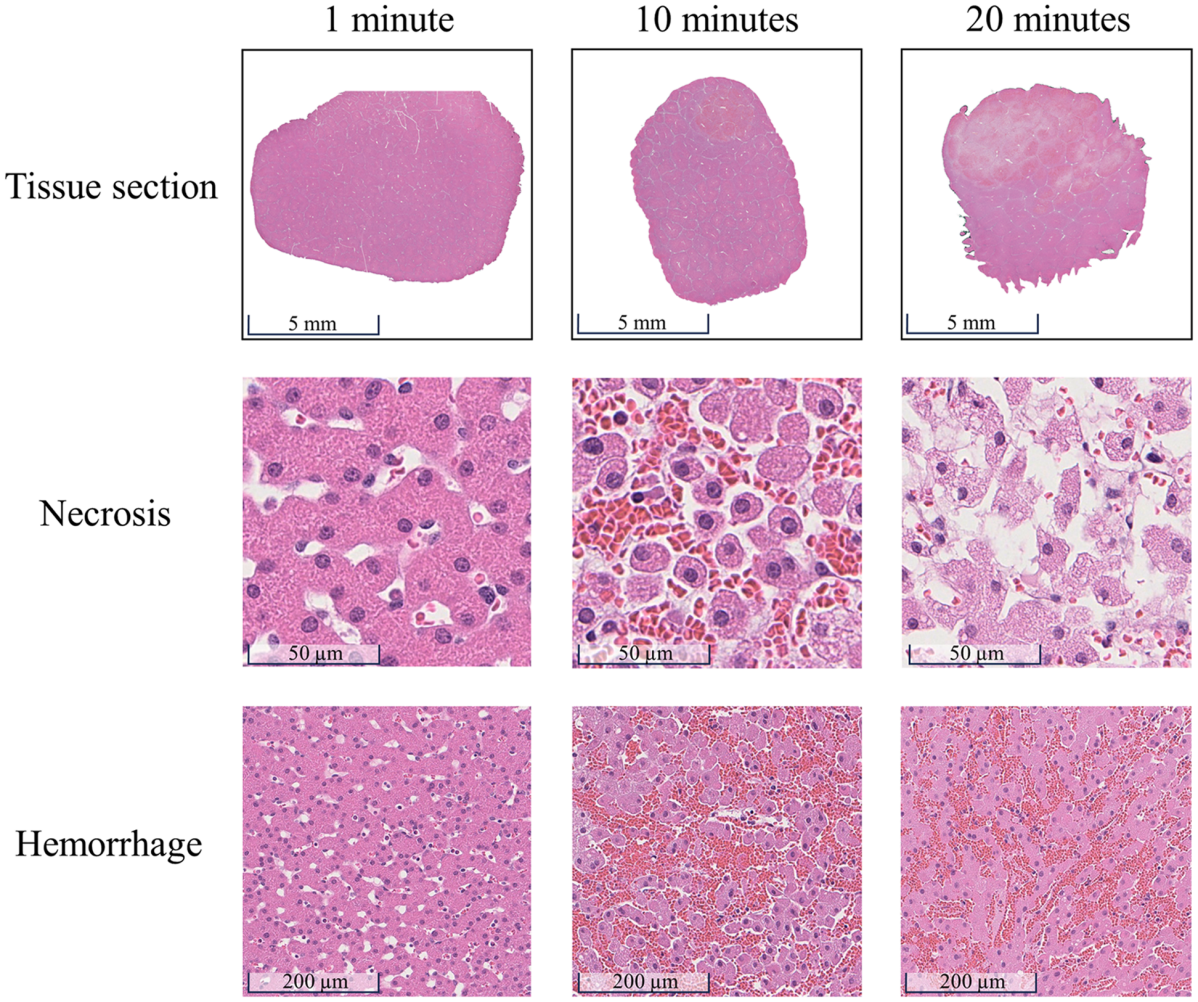
H&E sections from porcine liver samples irradiated for 1, 10, and 20 min. Regions of interest within the irradiated region were selected to perform necrosis and hemorrhage grading.

Figure 8 displays the characterization results of the H&E-stained sections. These sections were extracted at depths of 4  μm below the most superficial section that fully displayed the irradiated region and its adjacent non-irradiated area (i.e., 4, 284, and 164  μm for the 1, 10, and 20-min irradiation times, respectively) to closely match the quantitative assessment depths described in Sec. 2.7. Necrosis was defined by the loss of both hepatocellular and structural integrity, characterized by the loss of cell nuclei, cellular fragmentation, and content leakage. Inflammation manifested as aggregates of leukocytes within tissue. Hemorrhage was defined as the extravasation of red blood cells into interstitial spaces between cells. Based on these criteria, the porcine liver sample irradiated for 20 min exhibited severe necrosis and severe hemorrhage, whereas the sample irradiated for 10 min displayed mild necrosis and severe hemorrhage. In contrast, the porcine liver sample exposed to 1-min irradiation showed no pathological conditions. Minimal to no inflammation was observed in the three samples, as no leukocyte clusters were seen.

**Fig. 9:**
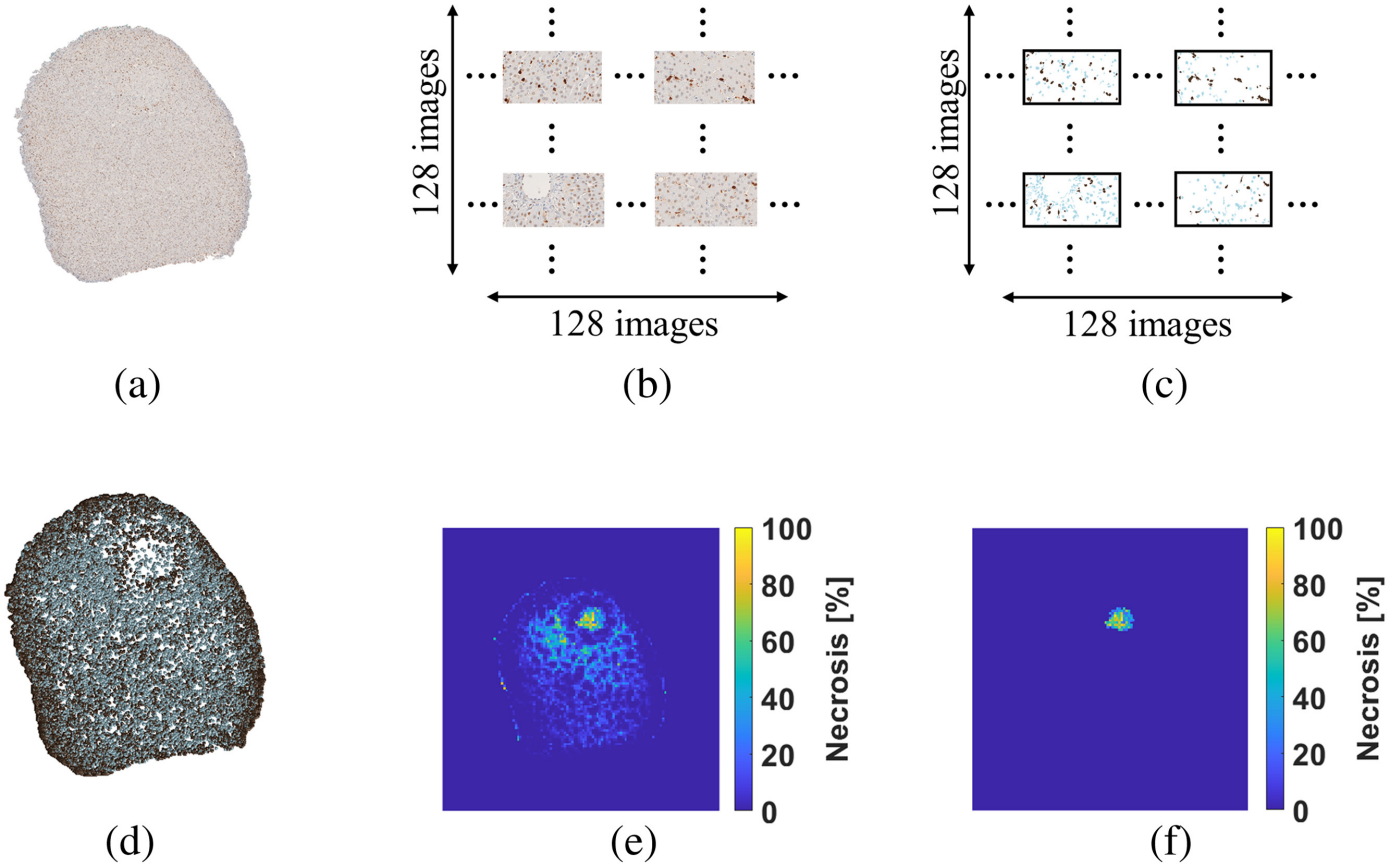
Image processing workflow for necrosis quantification of IHC sections: (a) IHC section, (b) mosaic of 16,384 RGB images, (c) segmentation, (d) blue and brown cells segmentation, (e) necrosis map, and (f) filtered map.

Figure 9 shows example results from the digitized IHC section processing pipeline. A representative IHC section from a swine liver sample irradiated for 10 min [Fig. 9(a)] was digitized and subsequently divided into a mosaic composed of 16384 JPEG images [Fig. 9(b)], and individual JPEG images were segmented [Fig. 9(c)]. These JPEG images were segmented with acceptable performance by the Attention U-Net (i.e., 0.97 DSC, 0.94 IoU, 0.99 recall, and 0.95 precision). The arrangement of the segmentation masks [Fig. 9(d)] provided a clearer image of the spatial distribution of blue and brown cells within the section. The overall cell density inside the irradiated area was notably lower compared to external areas. In addition, blue and brown cells were present throughout the section, whereas the presence of brown cells was reduced within the irradiated area. This segmentation example confirms the features observed during visual inspection of the IHC sections. The local necrosis map [Fig. 9(e)] computed using Eq. (6) exhibited a rounded central region with the highest necrosis percentage (i.e., mean percentage of 48.45%), surrounded by two scattered regions of lower and variable necrosis percentage (e.g., 28.11% and 35.44% mean percentage per independent region, resulting in an unrealistic combined necrosis percentage exceeding 100%, which leads to their classification as artifacts). The necrosis values corresponding to the irradiated region were preserved after filtering the artifacts [Fig. 9(f)].

**Fig. 10:**
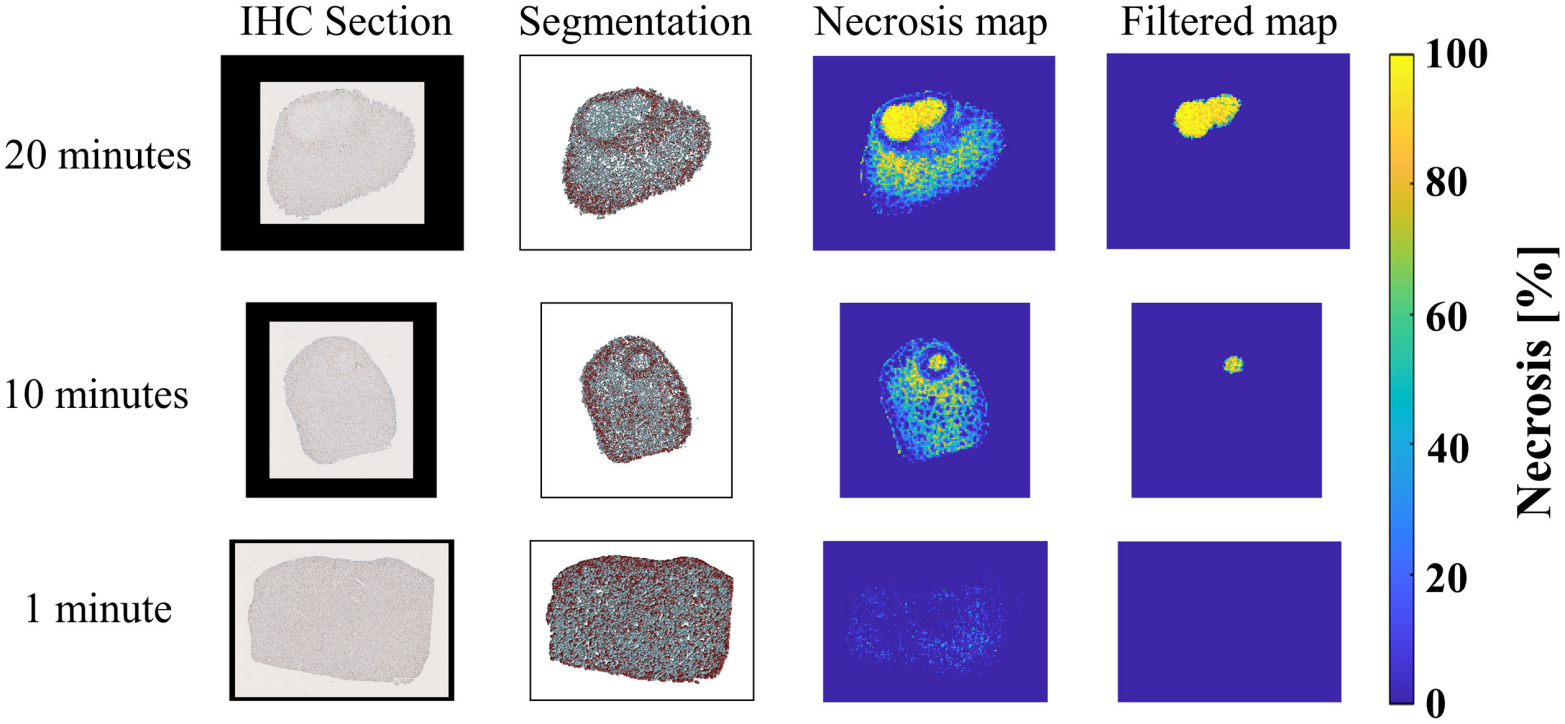
IHC section, blue and brown cells segmentation, initial necrosis map, and filtered necrosis map for three swine liver samples.

Figure 10 shows the necrosis maps from the irradiated liver samples, each extracted from a depth of 440  μm below the tissue surface. Laser application for 20 min caused complete disruption of cells in the illuminated area, yielding a mean necrosis percentage of 85.12%. Laser application for 10 min caused moderate disruption of cells in the illuminated area, resulting in a mean necrosis percentage of 65.49%. There are no signs of cell disruption when the laser is applied for 1 min, yielding 0% necrosis percentage in the irradiated region.

**Fig. 11:**
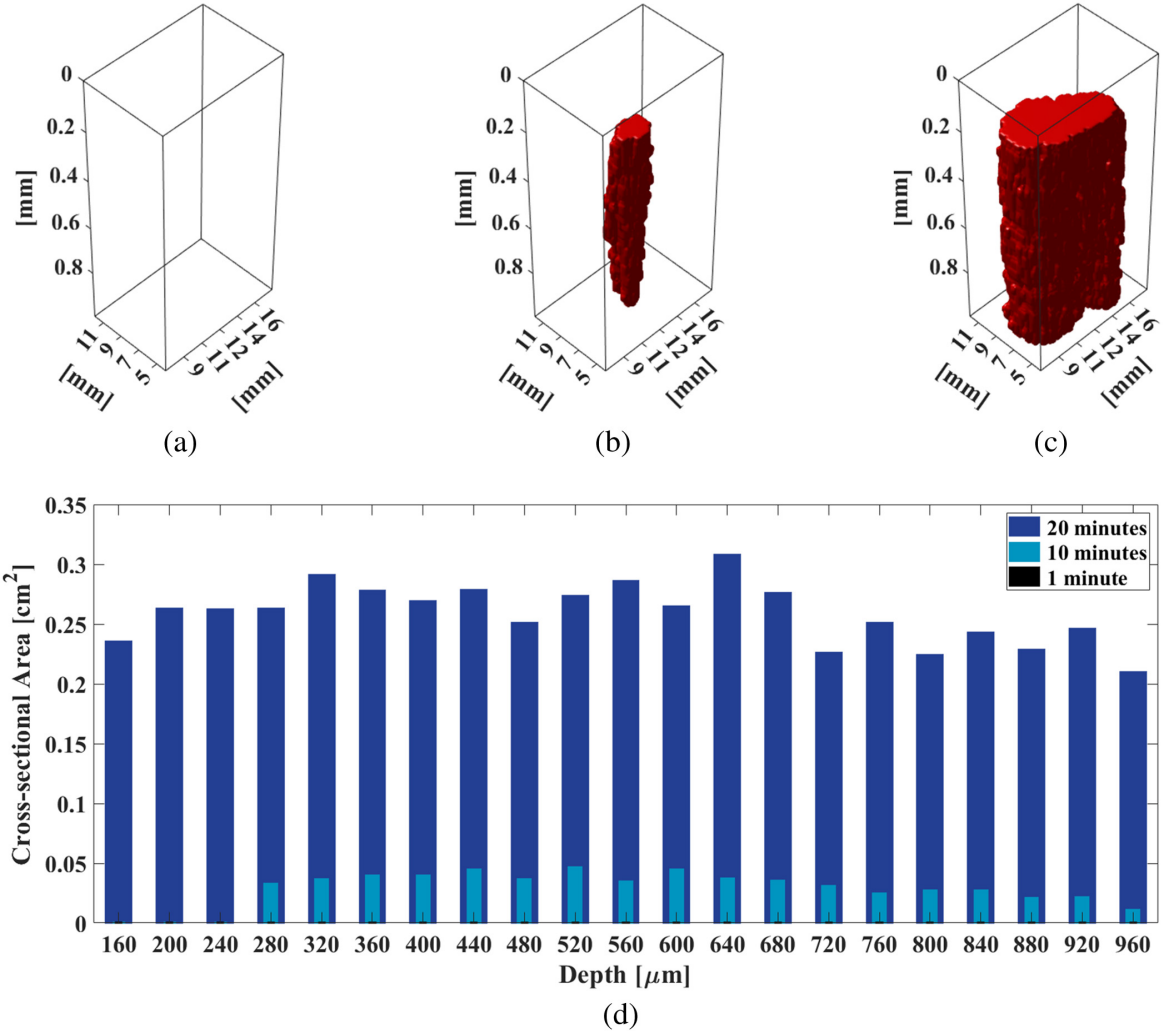
Volumetric necrosis reconstruction for swine liver samples irradiated for (a) 1 min, (b) 10 min, (c) 20 min, and (d) cross-sectional necrosis area as a function of tissue depth and laser duration.

Figure 11 shows the results obtained when quantifying the volume and area of necrotic cells as a function of depth from the irradiated surface. In Figs. 11(a)–11(c), the necrosis volumes measured 0, 3.27, and $24.68\;{\mathrm{mm}}^{3}$ after 1, 10, and 20 min of irradiation, respectively. In Fig. 11(d), the cross-sectional necrotic area remained generally constant and large (0.21 to $0.24\;{\mathrm{cm}}^{2}$) within the analyzed depth range (1 mm of swine liver tissue) for a 20-min exposure time. When the exposure time was shortened to 10 min, the cross-sectional necrotic area decreased at shallow depths (i.e., $0.03\;{\mathrm{cm}}^{2}$), then further decreased to $0.01\;{\mathrm{cm}}^{2}$ at a depth of 960  μm. No irradiation effects were observed with the 1-min laser irradiation time.

**Fig. 12:**
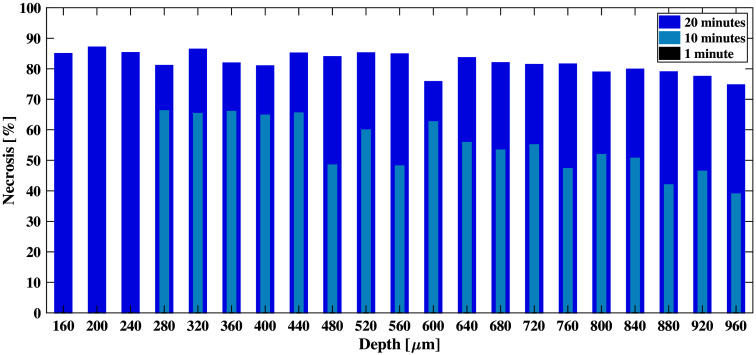
Necrosis percentage as a function of tissue depth and laser duration.

Figure 12 shows necrosis percentage as a function of depth for varying experimental laser exposure times. With a 20-min exposure time, the measured necrosis percentage ranged from 77.69% to 87.06%. When the exposure time was decreased to 10 min, the necrosis percentage ranged 38.95% to 66.22% for a similar mean energy of 73 mJ (see Fig. 1 and Table 2). There was no necrosis for the 1 min exposure time. When compared to the simulated results in Table 3 (obtained at a depth of 0  μm), the experimental results obtained at depths of 0, 280, and 160  μm for 1, 10, and 20-min irradiation times, respectively (due to the curvature of the experimental tissue samples, as noted in Sec. 2.7) revealed 0%, 66.22%, and 84.94% tissue necrosis, respectively, whereas the simulation framework predicted 1.23%, 66.23%, and 76.84%, respectively, which corresponds to deviations of 1.23%, 0.01%, and 8.1%, respectively. Therefore, the overall experimental deviation from predicted values range 0.01% to 8.1%. The experimental results obtained at depths of 280 to 320  μm and 160 to 200  μm for 10- and 20-min irradiation times, respectively, yielded mean ± one standard deviation tissue necrosis percentages of 61.24±4.49% and 86.38±1.15%, respectively. Therefore, the 0.01% to 8.1% deviations predicted with the simulation framework are within 1 to 2 standard deviations of the variations obtained over a minimal depth of 40  μm.

Overall, our empirical results demonstrate that liver imaging with no necrosis can be achieved with 73 mJ laser energy ($371.79\;\mathrm{mJ}/{\mathrm{cm}}^{2}$ fluence) applied for 1 min, with a laser wavelength of 750 nm, 5 ns pulse duration, and 10 Hz pulse repetition frequency. However, measurable necrosis was observed for exposure durations of 10 and 20 min, which is not considered safe under the same laser conditions (73 mJ energy, 750 nm, 5 ns pulse duration, and 10 Hz pulse repetition frequency). The 0.01% to 8.1% agreement between quantitative experimental results and simulation results, combined with ≤4.49% standard deviations on the quantitative measurements and the consistency between quantitative experimental results and qualitative outcomes from H&E staining collectively demonstrate the accuracy of our approach.

## 4 Discussion

This study is the first to quantify the thermal effects of laser exposure to *in vivo* liver tissue with 0.01% to 8.1% simulated necrosis prediction deviations from experimental results. Immunohistochemistry was successfully employed to provide the first known quantitative necrosis assessment across multiple depths and laser application time durations (Fig. 12), enabled by the exponential damage model introduced in Eqs. (6)–(8). These results are promising to provide tissue-specific MPE guidelines to maintain healthy liver tissue during laser-based optical and photoacoustic surgeries and interventions. In addition, the presented simulation framework and corresponding experimental protocols may be applied to other organs to achieve similar benefits.

Our primary simulation objective is to use simulated outputs to determine the threshold of predicted necrosis percentage indicative of visible liver tissue necrosis onset (although the predicted necrosis percentage provided by the simulation framework may not be exactly equivalent to the visible liver tissue necrosis observed in H&E-stained sections). When implementing the simulation framework, Table 3 indicates that up to 15.05% predicted necrosis will not produce visible liver tissue necrosis with H&E stains, while at least 66.23% predicted necrosis produces visible necrosis. It is reasonable to assume that the damage threshold for liver tissue resides within this range (i.e., 15.05% to 66.23%). In addition, the quantitative IHC results indicate that 0% necrosis occurred below this range (which supports the H&E observations). When the predicted necrosis at the tissue surface was above or equal to the upper limit of this range (i.e., ≥66.23%), the quantitative IHC results (Fig. 12) demonstrate that 38.95% to 87.06% necrosis occurred at multiple tissue depths and laser time durations (which also supports the H&E observations reported in Table 3). Therefore, the quantitative IHC results support our conclusions about the 15.05% to 66.23% predicted necrosis range wherein the damage threshold likely resides.

Although damage overestimation seems to have occurred with the simulation framework when compared to the quantitative IHC results, particularly when no necrosis was visibly detected (i.e., 1.23% deviation), experimental IHC results are generally consistent with the H&E results, as noted above. In addition, the simulated temperature probe was placed at the tissue surface, whereas the samples for the quantitative IHC comparison were taken from 0, 280, and 160  μm depths below the tissue surface for 1, 10, and 20-min irradiation times, respectively, as described in Sec. 2.7. While the unavoidable depth mismatch in the 10 and 20 min cases is an additional potential source of the reported discrepancy, the discrepancy was largest well beyond the range of the assumed damage threshold, and our ultimate goal is to develop standards for safe laser application and associated image guidance technology that will ideally avoid approaching tissue damage thresholds. It is promising that the simulation framework can predict both damaging necrosis percentages and negligible necrosis that is not sufficiently extensive to damage tissue, based on the qualitative and quantitative empirical H&E and IHC results in Figs. 8 and 12, respectively.

Additional confounding factors that could potentially impact experimental outcomes include the anatomical location of samples from the same organ and the spatial variation of optical and thermal parameters. However, our study analyzed three liver samples excised from the left lateral liver lobe of the same porcine specimen to draw our final conclusions. As these three samples were derived from a shared anatomical environment, is it reasonable to assume that most optical and thermodynamic parameters remained consistent across all specimens. Following the same argument, arterial blood temperature and density gradients can also be regarded as minimal.

Caspase-3 activation is generally associated with cell apoptosis. In contrast, necrosis is characterized in negative terms by the absence of caspase activation.^26^ Caspase-3 showed the presence of apoptotic cells surrounding the laser-exposed region and the absence of apoptotic cells within the laser-exposed region, indirectly identifying necrotic areas. While there are no conclusive reports elucidating the initiation of cellular apoptosis in the context of laser-induced injuries,^27^ there are three potential biological explanations. First, when living organisms face stress conditions, the synthesis of most proteins is suppressed. However, a group of highly conserved proteins called heat shock proteins are rapidly synthesized. In general, these proteins effectively inhibit apoptosis.^28^ Nevertheless, under specific circumstances such as prolonged stress conditions, the role of these proteins in caspase activation becomes deregulated.^29^ Second, cell damage caused by sudden shocks, such as radiation or heat, initially induces cellular swelling.^30^ Prolonged laser exposure can exacerbate this initial cell swelling by inducing mitochondria injury, promoting the release of mitochondrial proteins, such as cyto c, that trigger the activation of several caspase proteases.^31^ Third, disruption of the endoplasmic reticulum (ER), a critical organelle for cellular activities and survival, can contribute to the observed phenomenon. Stress conditions that impair the normal functioning of the ER result in the accumulation of unfolded proteins. If the stress persists and protein aggregation is persistent, signaling pathways transition from pro-survival to pro-apoptotic.^32^

One limitation of our study is that the presented empirical equations for necrosis quantification [i.e., Eqs. (6)–(8)] were proposed based on IHC sections for a cleaved Caspase-3 dilution of 1:1000. Changing the dilution factor may reduce the signal intensity or introduce background noise by exacerbating non-specific bindings, which may impact the applicability of our equations. Another limitation is that damage was produced in only two samples (quantified in 10 shallow sections total for comparison with simulations) out of the three samples reported herein and out of the 41 samples previously reported (including six control samples).^5^ While 10 sections may seem like a small sample size for comparison with damage prediction simulation results, there is agreement within 1-2 standard deviations, and ultimately our goal is to cause no harm (i.e., avoid laser-related damage) with our imaging technology. Therefore, the number of damaged samples out of 44 samples combined is considered as a positive attribute for photoacoustic technology, because the majority of samples did not show signs of damage and agree with simulation predictions.

Based on the totality of the simulation results herein and the experimental results in this publication and additional publications on this topic,^5^^,^^6^ we conclude that liver imaging with 750 nm laser wavelength, 5 ns pulse duration, 10 Hz pulse repetition frequency, and 30 mJ of laser energy emitted from a 5-mm diameter source (i.e., $152.79\;\mathrm{mJ}/{\mathrm{cm}}^{2}$ fluence) is safe when applied for at least 20 min, while 73 mJ (i.e., $371.79\;\mathrm{mJ}/{\mathrm{cm}}^{2}$ fluence) causes minimal (1.23% *in silico*, 0% *in vivo*) necrosis when applied for 1 min. Otherwise, the remaining time points (i.e., 10 and 20 min) should be avoided with 73 mJ energy, due to the 66.23% to 76.84% necrosis *in silico* and corresponding 66.22% to 84.94% necrosis measured *in vivo*. Future work will expand our experimental method and matching *in silico* model to determine safety with other tissues and tissue parameters that differ from the liver tissue validated and studied herein. We will additionally investigate motion-based methods to alleviate potential damage.^33^

## 5 Conclusion

This study introduces an innovative simulation framework to provide numerical estimations of laser-related tissue damage. We demonstrated the capabilities of integrating Monte Carlo optical simulations and COMSOL thermodynamic modeling to monitor the thermal impact of laser delivery across varying time intervals. The simulated predictions are well aligned with the experimental validation results. In addition, the temperature progression over time enabled the identification of critical time points related to important thermal processes, providing relevant insights into the status of the tissue of interest. Notably, our findings support safe photoacoustic liver imaging with a 5-mm diameter source emitting 750 nm wavelength laser light (with 5 ns pulse duration and 10 Hz pulse repetition frequency) when employing approximately 30 mJ of laser energy ($152.79\;\mathrm{mJ}/{\mathrm{cm}}^{2}$ fluence) with an imaging time ≤20  min. If opting for a higher energy of 73 mJ ($371.79\;\mathrm{mJ}/{\mathrm{cm}}^{2}$ fluence) for any reason (although this particular energy is not necessary for photoacoustic imaging), the laser application time should not exceed 1 min, as the next available validated temporal data point at this energy level (i.e., 10 min) causes severe damage. The presented approach and associated outcomes are promising for the introduction of tissue-specific safety guidelines for photoacoustic imaging and other optics-based imaging technologies that are designed to maximize signal-to-noise ratios while being designated as safe for patient use.

## Data Availability

Tabulated data to recreate relevant figures in the paper are publicly available at https://gitlab.com/pulselab/liverlasersafety. Additional data and code related to the paper will be publicly available whenever possible utilizing the above repository.
